# Directly Observed Care: Crossing the Chasm of Quality Measurement

**DOI:** 10.1007/s11606-022-07781-1

**Published:** 2022-09-20

**Authors:** A. Taylor Kelley, Saul J. Weiner, Joseph Francis

**Affiliations:** 1 Vulnerable Veteran Innovative Patient Aligned Care Team (VIP) Initiative, Informatics, Decision-Enhancement, and Analytic Sciences (IDEAS) Center, VA Salt Lake City Health Care System, Salt Lake City, UT USA; 2 Division of General Internal Medicine, Department of Internal Medicine, University of Utah School of Medicine, 30 North 1900 East, Room 5R218, Salt Lake City, UT 84132 USA; 3 Program for Addiction Research, Clinical Care, Knowledge and Advocacy (PARCKA), Division of Epidemiology, Department of Internal Medicine, University of Utah School of Medicine, Salt Lake City, UT USA; 4 Center of Innovation for Complex Chronic Healthcare, Jesse Brown VA Medical Center, Chicago, IL USA; 5 Division of Academic Internal Medicine and Geriatrics, Department of Medicine, University of Illinois-Chicago, Chicago, IL USA; 6 Office of Analytics and Performance Integration, Veterans Health Administration, Washington, DC USA

**Keywords:** directly observed care, healthcare quality, unannounced standardized patients, patient-centered care

## Abstract

After more than two decades of national attention to quality improvement in US healthcare, significant gaps in quality remain. A fundamental problem is that current approaches to measure quality are indirect and therefore imprecise, focusing on clinical documentation of care rather than the actual delivery of care. The National Academy of Medicine (NAM) has identified six domains of quality that are essential to address to improve quality: patient-centeredness, equity, timeliness, efficiency, effectiveness, and safety. In this perspective, we describe how directly observed care—a recorded audit of clinical care delivery—may address problems with current quality measurement, providing a more holistic assessment of healthcare delivery. We further show how directly observed care has the potential to improve each NAM domain of quality.

Two decades ago, the National Academy of Medicine (NAM) released a landmark report that provocatively described a gap between “the care we have” and “the care we could have” as a quality “chasm.”^1^ Quality measurement organizations then in their infancy, including the National Quality Forum (NQF) and National Committee for Quality Assurance (NCQA), responded swiftly, ushering in a rapid proliferation of quality measures to capture the structures, processes, and outcomes of clinical care. Today, the National Quality Forum (NQF) endorses more than 440 quality measures for use in healthcare settings, while similar measures in the NCQA’s Healthcare Effectiveness and Information Set (HEDIS) are used in health plans covering more than 191 million individuals.^2^

Surprisingly, none of these measures attempts direct observation of clinical care delivery. Instead, each relies on surrogates—provider self-report, electronic medical records, billing claims, or patient survey—that are steps removed from the actual clinical encounter. This limited understanding about the “care we have” curtails our ability to cross the quality chasm.

The problem is two-fold: first, surrogate measures are inaccurate and incomplete. When compared to direct audiovisual assessment of patient care, provider documentation in the medical record reveals substantial inaccuracies, both of commission (services charted but not delivered) and omission (services delivered but not charted).^3,4^ Claims data are similarly lacking (e.g., measuring whether blood pressure monitoring occurred but *not* whether it was taken correctly). Further, while patient surveys may capture perceptions of care, such as how well a provider listens, they cannot discern whether the provider acts appropriately on the information presented.

Second, clinicians are rewarded more for how well they document than how well they care for patients. In current practice, quality measures based on documentation alone generally fail to capture provider behaviors that are important to quality, such as appropriately utilizing motivational interviewing to facilitate lifestyle change or taking time to find out why their patients are not taking their medication as directed. By contrast, providers may document actions to reflect high-quality care they are expected to deliver but in fact either deliver them inadequately or not at all. Unfortunately, research using observers or incognito standardized patients demonstrates that the latter scenario is all too common.^3,4^ Such lapses in quality cannot be addressed using current measures of quality assessment.

This chasm between care as currently measured and the excellence we aspire to achieve calls for a transformative strategy that encompasses holistic measurement *throughout* the care cycle: before, during, and after a clinical encounter. Directly observed care—a recorded audit of care provided to real or standardized patients—enables such a quality measurement strategy (see Fig. 1).^5–8^ As Fig. 1 illustrates, directly observed care complements asynchronous measures of quality that occur before and after care delivery by providing synchronous data of actual care the patient receives. Although directly observed care draws upon evaluation techniques such as “mystery shoppers” initially found in hospitality industries and the social sciences, it is increasingly common in healthcare evaluation and research.

**Fig. 1:**
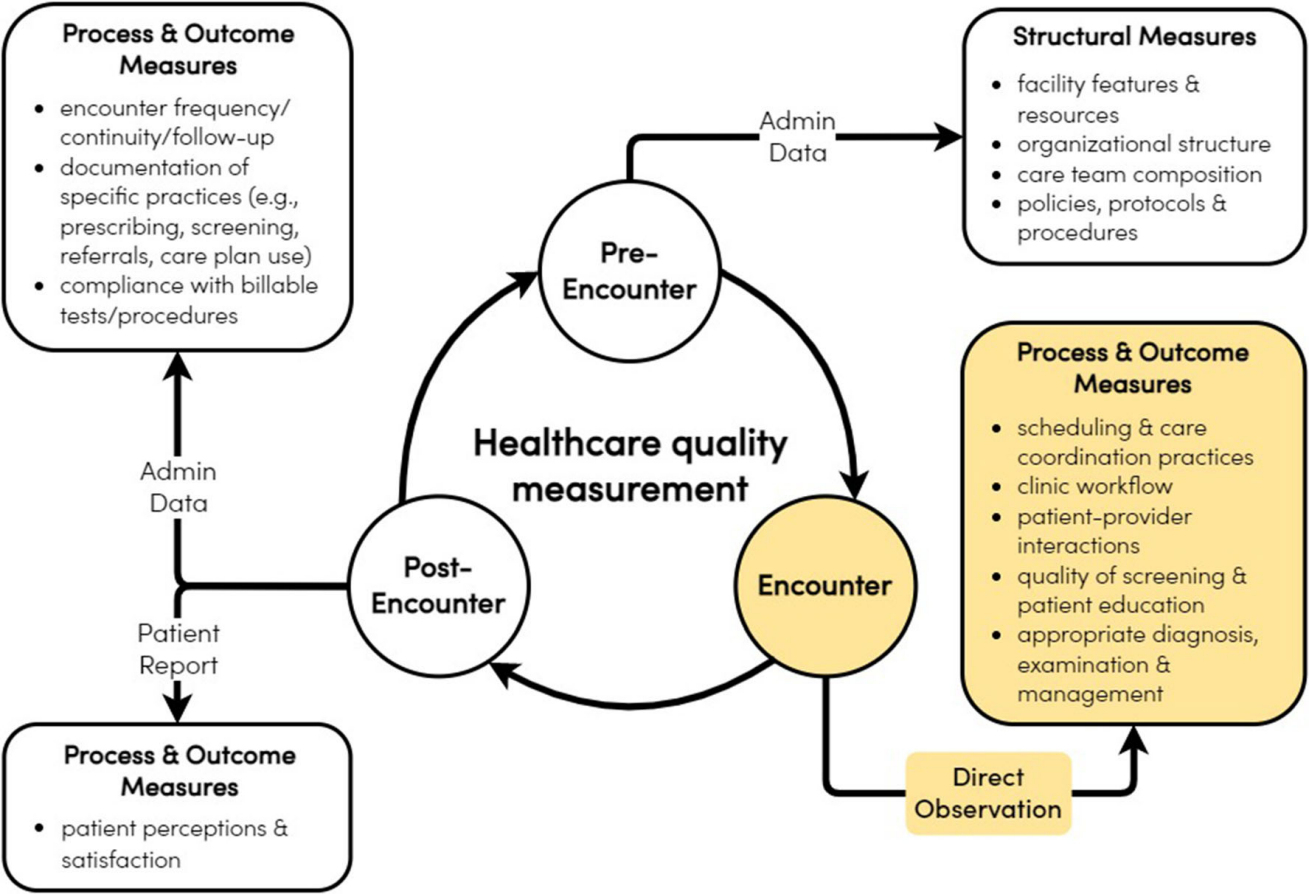
Holistic quality measurement throughout the cycle of healthcare delivery.

The potential of directly observed care to transform healthcare quality has implications for each quality domain identified in the NAM report—patient-centeredness, equity, timeliness, efficiency, effectiveness, and safety. We describe how each may benefit from this approach.

## PROMOTING PATIENT-CENTEREDNESS

Current quality measures can promote a “one size fits all” approach, inattentive to evidence that many aspects of care delivery require appropriate tailoring to each patient’s values and preferences.^9^ Knowing whether such attention was given requires observing the interaction between the clinician and patient. Did the physician ask the patient which treatment option they prefer after describing the risks and benefits? Did they attempt to understand why the patient is not taking a critical medication as directed and attempt to intervene where feasible? Directly observed care instead evaluates patient-centeredness by objectively capturing whether relevant patient life circumstances are elicited, recognized, and subsequently addressed within the clinical encounter—a predictor of healthcare outcomes.^10^ Professionalism, communication skills, and the use of stigmatizing language can likewise be evaluated.^11^ As shown in Fig. 1, these data complement patient experience data, medical records, and claims to create a 360-degree assessment of the entire care cycle.

Directly observed care can also reveal relative effectiveness of various practice patterns in delivering patient-centered care. For example, a meta-analysis of directly observed care across three datasets showed that patient concerns identified by providers are more likely to be incorporated into care plans when they are actively elicited by a provider rather than spontaneously stated by the patient.^12^ In addition, directly observed care allows for intrinsic risk adjustment by incorporating individual patient characteristics into quality measurement—a major challenge using claims data alone.^13^

## ENSURING EQUITY

Secret shoppers have played a pivotal role in studies of racism, including disparities in hiring practices, lending, and property rentals and sales.^14^ Unannounced standardized patients (USPs) may likewise serve an equally powerful role in identifying disparities in care at the level of the individual provider or health system. The advantage of evaluating healthcare equity through direct observation with USPs is that it avoids selection bias and confounding present in retrospective data while isolating one or more independent variables of interest (typically patient characteristics or concerns). Unbiased data are particularly helpful for provider feedback and subsequent evaluations that follow quality improvement interventions. For example, after a USP study of US Veterans experiencing homelessness showed that skin color predicted barriers to homeless services at community-based organizations, data feedback to these organizations resulted in resolution of the disparity within two years.^15^

USPs can likewise promote equitable comparisons of care quality across heterogeneous practice settings through a single standardized patient profile, which effectively serves as a reference standard. Recent USP evaluations using this approach have exposed wide practice variation in the provision of treatment for opioid use disorder, including residential treatment and pregnancy.^16,17^ Data such as these are compelling because they represent “ground truth” about the patient experience, inform targeted quality improvement interventions, and establish a baseline for subsequent assessments.

## IMPROVING TIMELINESS AND EFFICIENCY

A major drawback of current quality measures is the time lag—typically 12 months or longer—that separates care delivery and quality reporting.^18^ These delays in feedback undermine principles of continuous quality improvement. In contrast, data collected from directly observed care and coded by trained staff can be shared with providers as feedback soon after an encounter. In one study, USPs showed that providers receiving training to engage patients in shared decision-making for prostate cancer screening decreased utilization of screening tests among patients with uncertain clinical benefit—all within a three-month evaluation period.^19^

While current quality measurement strategies engender inefficiencies and an increasing burden on healthcare providers,^20^ there is growing evidence that observation data may be captured efficiently and sustainably over time when initial content coding validation by USPs is followed by direct observation of real patients (e.g., patient-collected audio) in regular clinical encounters.^21,22^ These two methods of directly observed care are complementary: USPs can portray specific scripts (e.g., high-stakes clinical scenarios where good communication is vital to treatment effectiveness) to assess how clinicians perform; patient-collected audio, in which patients volunteer to record their visits for quality improvement purposes, can assess how providers perform across a range of typical clinical situations. The former provides an experimental method of quality assessment, while the latter is observational.

As additional directly observed care data from both USPs and real patients become available, “big data” approaches, such as automating content analysis using machine learning algorithms, may improve efficiency. Such efforts are already underway.

## INCREASING EFFECTIVENESS AND SAFETY

The NAM report emphasizes that quality measurement must ultimately lead to quality improvement without resulting in excessive costs or self-defeating inefficiencies in care delivery. Unfortunately, quality measurement development since the report’s release has been subject to administrative burden, limited capability to account for risk, and other implementation challenges.^20^ Currently available data are not only inadequate to assess many aspects of quality^3,4^ but are also often problematic for developing accurate, sustainable, and comprehensive quality measures that appeal across stakeholders, improve outcomes, and reduce costs. Directly observed care shows promise in generating effective performance data. For example, in the US Veterans Health Administration, a provider feedback intervention based on directly observed care delivery improved providers’ ability to identify and address contextualized patient risks. The result of the intervention was a significant reduction in avoidable hospitalizations, signaling safer delivery of care and saving millions of dollars in acute care expenses—more than a 70-fold return on investment.^21^

By its nature, directly observed care may be used in different environments, which can increase the effectiveness of quality measurement. For example, measuring care quality for substance use treatment within a rural American Indian community, while normally difficult for conventional quality measurement approaches, can be adapted to assess verbal cues, treatment plans, and cultural sensitivity pertinent to the environmental context.^23^ Such adaptations are enhanced when they rely on stakeholder input to ensure representativeness of quality measures and on continuous audit and feedback to assess and improve care.

## POTENTIAL CHALLENGES

As with any quality measurement or quality improvement strategy, directly observed care is not without challenges. Unannounced standardized patients are resource-intensive. However, as the studies we have described here demonstrate, very few clinical encounters are needed to adequately assess care quality using USPs. Further, with development of patient-collected audio, USPs may not be necessary for ongoing quality assessment and improvement, adding to cost savings and improving return on investment.^21^ There are also cultural concerns about the privacy of a provider-patient interaction that could lead to consternation about directly observing care. Fortunately, early evidence suggests that when audio-recordings of visits are used for coaching and quality improvement rather than punitive or remedial purposes, provider satisfaction is high and *increases* over time.^24^ Broad stakeholder input has also been shown to improve implementation and sustainability.^22^ Finally, there is a potential challenge that the Hawthorne effect could bias results when providers are aware they are being observed. While the Hawthorne effect has been demonstrated in many research settings, early results of directly observed care find similar patterns of care quality between studies when providers are aware they are being observed and when they are not aware.^7,21^ While more data is needed, these early results are reassuring that biases related to the Hawthorne effect are minimal in directly observed care.

## CROSSING THE CHASM

Two decades ago, the NAM envisioned a future that would carry healthcare safely across the quality chasm. But before we can effectively improve quality, we must first cross the chasm of quality measurement. Those who deliver healthcare—as dedicated and well-intentioned as they may be—are unlikely to perform at their best if evaluated primarily by what they document rather than by what they do. While logistical and cultural challenges remain in implementing directly observed care into current quality measurement, it should be noted that direct observation is widely employed in other industries and has been implemented within healthcare at substantial scale in piloted quality improvement programs (full disclosure: including by the Institute for Practice and Provider Performance Improvement, of which one of the authors, SJW, is a principal). Although beyond the scope of this piece, the barriers are surmountable, and the costs of maintaining the status quo are likely greater.

As quality measurement organizations, policy makers, and other stakeholders consider the future of measuring and improving the care we provide, they should leverage data throughout the care cycle, including directly observed care. Only then can we cross the quality chasm in decades to come.
